# A Camera-Based Multimodal Defect Sensing Framework for Substation Equipment Monitoring via Cross-Modal Feature Mapping

**DOI:** 10.3390/s26123935

**Published:** 2026-06-21

**Authors:** Ziquan Liu, Hai Xue, Chengbo Hu, Chao Wei, Can Zhang

**Affiliations:** 1 Electric Power Research Institute, State Grid Jiangsu Electric Power Co., Ltd., Nanjing 211103, China; xueh5@js.sgcc.com.cn (H.X.); huchengbodky@js.sgcc.com.cn (C.H.); weic1@js.sgcc.com.cn (C.W.); 2 Nanjing Power Supply Company, State Grid Jiangsu Electric Power Co., Ltd., Nanjing 210019, China

**Keywords:** substation equipment monitoring, multimodal perception, cross-modal feature mapping, advanced sensing technology, intelligent inspection, neuro-symbolic reasoning

## Abstract

**Highlights:**

**What are the main findings?**

- A multimodal defect sensing framework for substation equipment monitoring was developed by integrating vertical domain pre-training, bidirectional cross-modal feature mapping, and hierarchical neuro-symbolic reasoning.
- The proposed method achieved the best overall detection performance on the test set, reaching 90.8% mAP@0.5, 68.7% mAP@0.5:0.95, and 89.4% F1-score, while also reducing logically conflicting false positives through knowledge-guided reasoning.

**What are the implications of the main findings?**

- Cross-modal semantic alignment between inspection images and defect text can significantly enhance the perception of complex, weakly salient, and small-target defects in substation inspection scenarios.
- Incorporating topology knowledge and spatial rules into posterior reasoning improves the reliability, interpretability, and engineering applicability of intelligent inspection systems for equipment status monitoring and early warning.

**Abstract:**

To address the limitations of vision-only defect detection, image–semantic misalignment, and spatial-logic conflicts in complex substation inspection scenarios, this paper proposes a camera-sensor-based multimodal defect sensing framework with cross-modal feature mapping for substation equipment monitoring. The proposed framework integrates field inspection images acquired by camera sensors, defect textual descriptions, and equipment topology knowledge and establishes a unified domain-adaptive pre-training–bidirectional cross-modal mapping–hierarchical reasoning workflow. First, a Contrastive Language–Image Pre-training (CLIP)-based domain-adaptive pre-training strategy is developed to enhance the representation of equipment categories, defect attributes, and inspection-scene semantics. Second, a bidirectional cross-modal feature mapping network is constructed to model fine-grained interactions between candidate visual regions and textual semantics, where uncertainty-aware fusion and prototype constraints are introduced to improve semantic alignment and defect discrimination. Third, a hierarchical neuro-symbolic reasoning module incorporates equipment topology and spatial rules for posterior verification, logical consistency checking, and false-positive suppression. Experiments on a substation inspection image dataset demonstrate that the proposed method achieves 90.8% mAP@0.5, 68.7% mAP@0.5:0.95, and 89.4% F1-score, outperforming mainstream and recent detection models.

## 1. Introduction

Substations are key hubs for power collection, transformation, and distribution, and their equipment status directly affects power grid safety, stability, and reliability [1,2]. The development of new power systems, high-proportion renewable energy integration, bidirectional source–load interaction, and increased operational uncertainty have raised higher requirements for equipment monitoring, risk warning, and abnormal-event response. In recent years, fixed high-definition cameras, inspection robots, infrared thermography, edge-intelligent terminals, and intelligent sensing technologies have been increasingly used in substation inspection, promoting equipment monitoring toward online, automated, and intelligent operation. Among them, image-sensor-based defect perception has become an important research direction because of its high efficiency, low cost, and suitability for continuous monitoring [3,4].

Deep learning has promoted power equipment defect identification from experience-based judgment to data-driven recognition [5,6]. Existing studies show that convolutional neural networks and object detection frameworks can identify typical defects such as insulator damage, oil leakage, instrument faults, and component corrosion [7,8]. Detection methods for Unmanned Aerial Vehicle (UAV) inspection, robot inspection, and complex-scene image analysis have also progressed in small-target localization, feature enhancement, and lightweight deployment [9,10]. In addition, infrared thermography combined with visible-light detection provides richer information for thermal anomaly diagnosis, condition assessment, and risk perception [4,11,12,13].

However, real substation inspection scenarios remain complex. Substation equipment varies in type and layout, with significant differences in appearance, scale, and background environment [2,14]. Field images are also affected by illumination changes, weather disturbances, seasonal variations, occlusion, and cluttered backgrounds, resulting in weakly salient, small-scale, and low-contrast defects [14,15]. For complex defects such as bird nests, suspended foreign objects, loose components, and abnormal parts, single-modal visual appearance is still insufficient to ensure accuracy, robustness, and cross-scenario generalization [14,16].

To address these challenges, existing studies have explored object detection, image segmentation, anomaly identification, data augmentation, and multi-source information fusion [6,15,17]. Some methods improve detection performance by enhancing backbone feature extraction, optimizing multi-scale feature fusion, introducing semantic-enhanced anomaly learning, and conducting joint detection–segmentation modeling [14,15,16]. Other methods alleviate insufficient samples through synthetic data, adversarial generation, and foggy-scene construction [9,18]. For small and blurred targets, lightweight network design, semantic-enhanced detection, and multi-domain collaborative feature modeling have also been introduced to balance real-time performance and accuracy [10,15,16]. Nevertheless, most existing methods still focus mainly on single-modal visual information, while defect semantics, textual descriptions, equipment spatial relationships, and operation and maintenance rules remain insufficiently utilized [18,19].

Recent intelligent fault diagnosis studies have improved diagnostic reliability, label utilization efficiency, and cross-domain generalization through a lightweight multi-expert wavelet Transformer [20], entropy-oriented semi-supervised prototype contrastive learning [21], and spatial–channel collaborative multi-scale graph transfer learning [22]. These studies provide useful insights into trustworthy representation learning and knowledge transfer. However, they mainly focus on vibration, current, acoustic, or time-series signals, whereas substation visual defect perception relies on camera-sensor images and faces additional challenges such as small targets, complex backgrounds, image–text semantic inconsistency, and topological spatial constraints. Therefore, a camera-sensor-based multimodal perception method is needed to jointly model visual features, defect semantics, and operation and maintenance rules.

With the development of vision–language pre-trained models, cross-modal semantic modeling provides a new perspective for complex defect perception in substations. Vision–language models represented by CLIP can associate visual features with semantic descriptions through image–text alignment learning and have shown strong potential in few-shot, zero-shot, and open-set anomaly recognition tasks [23,24]. For substation equipment monitoring, unified modeling of equipment images, defect text descriptions, and domain-specific corpora can help overcome the limitations of single-modal visual representation. Recent studies on multimodal power sample feature transfer also show that cross-modal information decoupling and alignment can enhance complementary representation among heterogeneous information sources [23,25,26]. Meanwhile, neuro-symbolic learning, knowledge graph fusion, and knowledge-enhanced zero-shot reasoning provide methodological support for introducing equipment topology, spatial logic, and expert operation and maintenance knowledge [27,28]. By combining data-driven detection with knowledge-driven logical reasoning, the rationality, reliability, and interpretability of monitoring results can be further improved [27,28,29].

Based on the above analysis, this paper focuses on three key issues in complex substation defect monitoring: difficulty in multimodal semantic alignment, insufficient perception capability for complex defects, and lack of knowledge constraints in monitoring results. A multimodal defect perception method based on cross-modal feature mapping is proposed, and a unified vertical-domain pre-training–cross-modal mapping–hierarchical reasoning framework is constructed to realize collaborative modeling of visual information, textual semantics, and domain knowledge. The main contributions are as follows:

(1) A multimodal defect perception framework for substation image sensing data is proposed, covering field image acquisition, dataset construction, model inference, rule verification, and operation-and-maintenance-readable output.
(2) A vertical-domain adaptive pre-training module for the power industry is designed based on the CLIP framework to improve the representation capability for complex defects and few-shot scenarios.
(3) An image–text bidirectional cross-modal feature mapping network is constructed to enhance fine-grained semantic consistency between candidate visual regions and textual descriptions.
(4) A neuro-symbolic reasoning mechanism integrating equipment topology knowledge is introduced to verify logical consistency, constrain spatial relationships, reduce unreasonable false positives, and improve result reliability.

## 2. Sensor-Based Substation Defect Perception System and Data Acquisition

In actual intelligent substation inspection scenarios, equipment defect identification relies on field image acquisition, data transmission, model inference, and operation and maintenance feedback to form a continuous workflow. Based on this scenario, this section introduces the system architecture, field image acquisition, dataset construction, and operation-and-maintenance output from the perspective of sensing data processing.

### 2.1. Overall System Architecture

**Figure 1:**
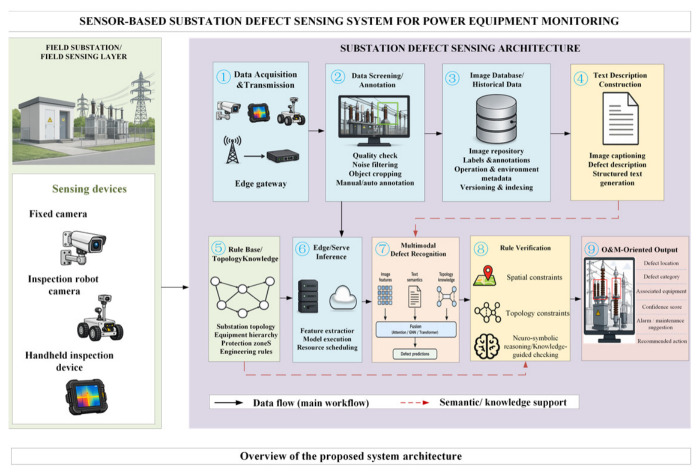
Overall Architecture of the Sensor-Based Substation Defect Perception System.

As shown in Figure 1, the system includes field image acquisition, data transmission, edge/server inference, multimodal defect identification, rule verification, and operation-and-maintenance-readable output.

After field images and auxiliary information are uploaded to the edge node or station-side server, the system performs preprocessing, candidate region generation, and inference. It then fuses image features, textual semantics, and topology knowledge for defect localization and classification, verifies candidates using spatial and topological rules, and generates defect explanations and alarm information.

From the perspective of system input and output, the defect perception process can be expressed as

(1) $$Y=F_{\Theta }(I,M,T,G)$$

where I denotes the inspection image set collected by field image sensors, M denotes the acquisition and contextual metadata associated with the inspection images, T denotes the defect text description set, G denotes equipment topology and operation–maintenance rule knowledge, Y denotes the defect perception output, and Θ denotes model parameters. The detailed metadata fields and camera-sensor configuration are further described in Section 2.2.

### 2.2. Field Image Acquisition and Sensor Configuration

In this study, the field image acquisition devices are treated as visible-light camera sensors. According to their deployment modes in substation inspection, they include fixed surveillance cameras, inspection-robot cameras, and handheld inspection cameras or mobile inspection terminals. These devices share the same basic sensing modality, namely Red, Green, and Blue (RGB) visible-light imaging, but differ in acquisition geometry, viewpoint stability, shooting distance, and disturbance sources. Fixed surveillance cameras are installed at typical monitoring points and provide relatively stable views of equipment regions. Inspection-robot cameras are mounted on mobile inspection platforms and acquire images from variable viewpoints during patrol tasks. Handheld inspection cameras or mobile inspection terminals are used by operators during manual inspection and review for supplementary image acquisition.

For the i-th field acquisition sample, its sensing data can be expressed as

(2) $$x_{i}=(I_{i},m_{i}),$$

where $I_{i}$ is an RGB image collected by a visible-light camera sensor, and $m_{i}$ denotes the corresponding acquisition metadata. The metadata include acquisition time, camera or device ID, inspection point, equipment bay or equipment region, target equipment ID, shooting angle or preset position, and inspection task information. The RGB image $I_{i}$ is used as the main input for model training and inference, while the metadata $m_{i}$ are used for sample archiving, equipment association, semantic-description construction, rule verification, and maintenance-oriented result interpretation.

The field images were collected from real substation inspection tasks rather than from a single laboratory imaging platform; therefore, the camera models and detailed imaging parameters were not fully identical across all samples. In practical inspection records, available camera-level information generally includes the original image resolution, image format, compression mode, capture frequency, focal length or zoom mode, field of view, exposure mode, installation or shooting distance, and acquisition viewpoint. Together with metadata such as acquisition time, camera/device ID, inspection point, equipment region, target equipment ID, and shooting angle or preset position, these records are used for sample archiving, equipment association, semantic-description construction, rule verification, and maintenance-oriented result interpretation.

Camera-based image acquisition in field environments is inevitably affected by sensor- and scene-related uncertainties, such as exposure fluctuation, sensor noise, motion blur, defocus, lens distortion, compression noise, viewpoint variation, illumination change, weather disturbance, occlusion, and background clutter. These factors may affect image quality, defect texture, boundary clarity, target scale, and spatial context and may further propagate to preprocessing, feature extraction, candidate localization, cross-modal semantic matching, and final identification. For example, motion blur or defocus may weaken local defect features, lens distortion or viewpoint variation may affect bounding-box localization, and illumination or background changes may reduce the reliability of image–text semantic alignment. The image quality screening, data augmentation, environmental consistency regularization, and rule-based posterior verification used in this work can improve robustness to some extent, but they should be regarded as robustness-enhancement strategies rather than physical calibration or complete correction of stochastic camera-sensor errors. Therefore, the reported results reflect dataset-based validation under the covered inspection conditions, rather than guaranteed long-term online accuracy under continuously changing field conditions.

### 2.3. Dataset Construction and Annotation

The field-collected images are screened by removing or marking duplicate, blurred, overexposed, underexposed, target-invisible, and invalid-background images. Images with occlusion, strong illumination, fog, small targets, or complex backgrounds are retained if confirmed by experts.

The screened images are organized by equipment type, inspection area, and normal/abnormal status. Defect samples are annotated with bounding boxes and categories, normal samples are labeled as normal, and all annotations are expert-reviewed. The image dataset can be expressed as

(3) $$D={\left\{(I_{i},y_{i},B_{i})\right\}}_{i=1}^{N}$$

where N=11,842, $y_{i}\in 0,1$ is the sample status label, $y_{i}=0$ denotes a normal sample, and $y_{i}=1$ denotes a defective sample; $B_{i}$ denotes the set of defect bounding boxes. For a normal sample, $B_{i}=\emptyset$.

In addition to image annotation, this paper constructs defect text descriptions and equipment knowledge rules. Text descriptions are generated from defect categories, equipment attributes, spatial locations, and status information for image–semantic alignment. Equipment rules are built from topology, typical defect locations, and operation and maintenance experience for posterior logical verification. Details of data scale, text descriptions, rules, and dataset split are provided in the experimental section.

### 2.4. Task Definition and Operation-and-Maintenance Output

This paper focuses on equipment defect perception using substation image sensing data. Given inspection images and related semantic and rule information, the model performs defect localization, category identification, and consistency verification and outputs structured results for operation and maintenance personnel. Unlike traditional object detection, this task further considers defects, equipment objects, spatial locations, and operation and maintenance rules to improve interpretability and engineering usability.

For the input image $I_{i}$, the final output is denoted as

(4) $$Y_{i}={\left\{(b_{i,k},c_{i,k},p_{i,k},e_{i,k},r_{i,k},a_{i,k})\right\}}_{k=1}^{K_{i}}$$

where $b_{i,k}$ denotes the bounding box of the k-th defect target, $c_{i,k}$ denotes the defect category, $p_{i,k}$ denotes the detection confidence, $e_{i,k}$ denotes the associated equipment or component, $r_{i,k}$ denotes the rule verification result, $a_{i,k}$ denotes the warning explanation or disposal suggestion provided for operation and maintenance personnel, and $K_{i}$ denotes the number of candidate defect targets detected in image $I_{i}$.

This paper focuses on equipment defect perception using substation image sensing data. Given inspection images and related semantic and rule information, the model localizes defects, identifies categories, verifies consistency, and outputs structured results for operation and maintenance personnel. Unlike traditional object detection, this task further considers equipment objects, spatial locations, and operation and maintenance rules to improve interpretability and engineering usability.

## 3. Multimodal Defect Perception Method

This paper proposes a multimodal defect perception method for substation equipment identification. Using visible-light images, defect descriptions, and topology rules, the method achieves defect localization, semantic recognition, and logical consistency verification through image–text pre-training, cross-modal mapping, prototype constraints, and knowledge rule verification.

### 3.1. Overall Framework of the Proposed Method

**Figure 2:**
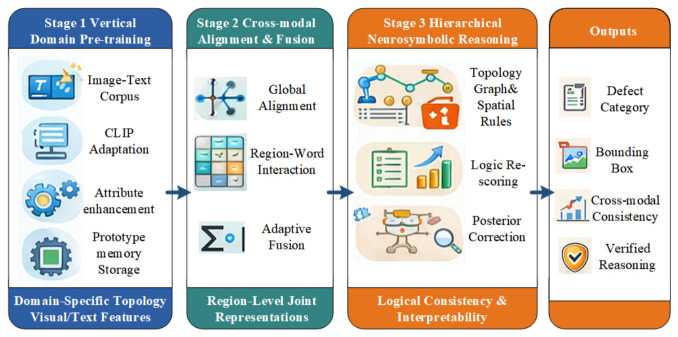
Framework of the proposed multimodal defect sensing method.

As shown in Figure 2, the proposed framework consists of four modules: vertical-domain image–text pre-training for semantic representation enhancement, bidirectional cross-modal feature mapping for fine-grained region–text correspondence, prototype-constrained defect discrimination for similar-category separation, and knowledge rule verification for posterior re-scoring, false-positive reduction, and interpretability improvement.

### 3.2. Vertical-Domain Image–Text Pre-Training

Substation inspection images involve diverse equipment, complex backgrounds, and strong defect-semantic dependence. Since general vision–language models lack power-equipment and operation–maintenance knowledge, this paper constructs a vertical-domain image–text pre-training module to adapt the visual and text encoders using inspection images and multi-granularity defect descriptions.

Given an image sample $I_{i}$ and its corresponding text description $T_{i}$, the visual encoder $E_{v}(\cdot )$ and the text encoder $E_{t}(\cdot )$ extract normalized features, respectively:

(5) $$v_{i}=\frac{E_{v}(I_{i})}{\|E_{v}(I_{i}){\|}_{2}},\;t_{i}=\frac{E_{t}(T_{i})}{\|E_{t}(T_{i}){\|}_{2}}$$

In a training batch, the similarity between image features and text features is defined as follows:

(6) $$S_{ij}=\frac{v_{i}^{T}t_{j}}{\tau }$$

where τ is the temperature coefficient. Through image–text contrastive learning, matched image–text pairs are pulled closer in the shared semantic space, while unmatched sample pairs are pushed farther apart. The corresponding bidirectional image–text contrastive loss is expressed as

(7) $$L_{itc}=-\frac{1}{B}\sum_{i=1}^{B}\left[\log\frac{\exp(S_{ii})}{\sum_{j=1}^{B}\exp(S_{ij})}+\log\frac{\exp(S_{ii})}{\sum_{j=1}^{B}\exp(S_{ji})}\right]$$

where B denotes the batch size. This loss can strengthen the semantic correspondence between inspection images and defect categories, defect attributes, spatial locations, and equipment status descriptions, thereby providing basic representations for subsequent cross-modal feature mapping. In addition to image–text contrastive learning, defect attribute supervision is further introduced to enhance the model’s representation ability for fine-grained defect features. Let the attribute label of the i-th sample be $a_{i}$, and the attribute prediction result be

(8) a^i=σ(Wavi+ba)

where σ(⋅) denotes the Sigmoid function, and $W_{a}$ and $b_{a}$ are the parameters of the attribute prediction head. The attribute supervision loss is defined as

(9) Lattr=−1B∑i=1B∑c=1Caai,cloga^i,c+(1−ai,c)log(1−a^i,c)

where $C_{a}$ denotes the number of attribute categories, and $a_{i,c}$ and a^i,c denote the ground-truth label and predicted probability of the i-th sample on the c-th attribute, respectively. This constraint enables the model not only to learn whether it belongs to a certain defect class but also to further focus on fine-grained attribute information such as breakage, corrosion, suspension, attachment, occlusion, and abnormal status.

To improve representation stability under illumination, weather, viewing-angle, and background variations, an environmental consistency constraint is introduced. For the same-equipment image pairs collected under different conditions or augmented by brightness perturbation, contrast variation, slight blurring, and occlusion simulation, the feature consistency loss is defined as

(10) $$L_{inv}=\frac{1}{B}\sum_{i=1}^{B}{\left\|v_{i}^{\left(1\right)}-v_{i}^{\left(2\right)}\right\|}_{2}^{2}$$

where $v_{i}^{\left(1\right)}$ and $v_{i}^{\left(2\right)}$ denote the image features obtained from the same equipment area under different acquisition conditions or different augmentation methods. It should be noted that augmented image pairs are mainly used for consistency regularization and cannot independently serve as sufficient evidence that the model adapts to all real weather and seasonal variations; robustness in real environments still needs to be experimentally verified using multi-scenario field images.

By integrating image–text contrastive learning, attribute supervision, and environmental consistency constraints, the vertical-domain pretraining objective is defined as

(11) $$L_{pre}=\lambda_{1}L_{itc}+\lambda_{2}L_{attr}+\lambda_{3}L_{inv}$$

where $\lambda_{1}$, $\lambda_{2}$, and $\lambda_{3}$ are weight coefficients. Through this pretraining process, the model can obtain image–text basic representations that are more suitable for substation inspection scenarios, thereby providing support for subsequent region-level cross-modal mapping and defect category discrimination.

### 3.3. Bidirectional Cross-Modal Feature Mapping

Vertical-domain image–text pretraining enhances global image–text semantic alignment, but substation defects are often local, small, and context-dependent, making global matching insufficient. Therefore, this paper constructs a bidirectional cross-modal feature mapping module to build fine-grained associations between local visual features and textual semantic units at the candidate-region level, improving recognition of complex and weakly salient defects. Let the candidate region features extracted by the visual detection backbone be

(12) $$V={\left\{v_{i}^{r}\right\}}_{i=1}^{N_{r}}$$

The set of word-level or phrase-level semantic features extracted by the text encoder is denoted as

(13) $$W={\left\{t_{j}^{w}\right\}}_{j=1}^{N_{t}}$$

where $N_{r}$ denotes the number of candidate regions, and $N_{t}$ denotes the number of textual semantic units. Candidate region features mainly describe the local appearance and spatial location of the image, while textual semantic features correspond to descriptive information such as defect category, attribute, location, and status.

First, visual-to-text mapping is used to supplement semantic descriptions for candidate regions. For the i-th candidate region, the matching weight between it and the j-th textual semantic unit is defined as

(14) $$\alpha_{ij}=\frac{\exp\left({\left(W_{v}v_{i}^{r}\right)}^{T}(W_{t}t_{j}^{w})\right)}{\sum_{j=1}^{N_{t}}\exp\left({\left(W_{v}v_{i}^{r}\right)}^{T}(W_{t}t_{j}^{w})\right)}$$

where $W_{v}$ and $W_{t}$ are learnable mapping matrices. Based on this weight, the text-enhanced regional semantic representation can be obtained as

(15) $${\overset{˜}{t}}_{i}=\sum_{j=1}^{N_{t}}\alpha_{ij}t_{j}^{w}$$

This means that the category, attribute, and location descriptions most relevant to the candidate region are introduced into the regional features, enabling the model to determine whether the candidate region matches the defect semantics.

Correspondingly, text-to-visual mapping is used to filter key visual regions according to textual semantics. For the j-th textual semantic unit, its attention weight for the i-th candidate region is defined as

(16) $$\beta_{ji}=\frac{\exp\left({\left(W_{t}^{\prime }t_{j}^{w}\right)}^{T}(W_{v}^{\prime }v_{i}^{r})\right)}{\sum_{i=1}^{N_{r}}\exp\left({\left(W_{t}^{\prime }t_{j}^{w}\right)}^{T}(W_{v}^{\prime }v_{i}^{r})\right)}$$

where $W_{t}^{\prime }$ and $W_{v}^{\prime }$ are reverse mapping matrices. The visual aggregation representation guided by textual semantics is expressed as

(17) $${\overset{˜}{v}}_{j}=\sum_{i=1}^{N_{r}}\beta_{ji}v_{i}^{r}$$

Visual-to-text mapping identifies the defect semantics of each candidate region, while text-to-visual mapping highlights regions relevant to given defect semantics. Together, they form region-level bidirectional semantic alignment and improve recognition of small, weak-texture, and semantics-dependent defects.

Based on the mapping results, a region-level cross-modal representation is constructed. For candidate region (i), its visual-dominated fused representation is defined as

(18) $$z_{i}^{v}=\phi_{v}\left(\left[v_{i}^{r},{\overset{˜}{t}}_{i},v_{i}^{r}⊙{\overset{˜}{t}}_{i},|v_{i}^{r}-{\overset{˜}{t}}_{i}|\right]\right)$$

where [⋅] denotes feature concatenation, ⊙ denotes element-wise multiplication, and $\phi_{v}(\cdot )$ is a multilayer perceptron mapping function. This representation simultaneously preserves visual appearance, textual semantics, modal interaction, and modal difference information.

Considering that different defects have different degrees of dependence on image and text information, this paper further introduces an uncertainty-aware gating mechanism to adaptively adjust the contributions of visual-dominated features and semantic-dominated features. Let $z_{i}^{v}$ and $z_{i}^{t}$ denote the visual-dominated and text-dominated cross-modal representations, respectively, and let $u_{i}^{v}$ and $u_{i}^{t}$ denote the uncertainty estimates of the two modalities, respectively. Then the gating weight is defined as

(19) $$g_{i}=\sigma \left(W_{g}\left[z_{i}^{v},z_{i}^{t},u_{i}^{v},u_{i}^{t}\right]+b_{g}\right)$$

The final region-level fused representation is expressed as

(20) $$z_{i}^{f}=g_{i}z_{i}^{v}+(1-g_{i})z_{i}^{t}$$

where a larger $g_{i}$ indicates that the model relies more on visual-dominated information, while a smaller $g_{i}$ indicates that the model relies more on textual semantic guidance. This mechanism enables the model to maintain image-dominated discrimination when visual features are clear and to introduce more semantic supplementation when the target is small, the texture is weak, or the background is complex, thereby improving the stability of defect perception in complex inspection scenarios.

### 3.4. Prototype-Constrained Defect Discrimination

Some substation defect categories show strong visual similarity and sample imbalance. For example, bird nests may resemble branch occlusion, suspended foreign objects may resemble background clutter, and surface abnormalities may resemble light reflection. Relying only on the detection head can cause unclear category boundaries or unstable few-sample discrimination. Therefore, this paper introduces prototype constraints, using textual and visual prototypes as category centers.

For the c-th defect category, the textual semantic prototype $p_{c}$ and the visual prototype $q_{c}$ are constructed:

(21) $$p_{c}=\frac{1}{\left|T_{c}\right|}\sum_{T\in T_{c}}E_{t}(T),\;q_{c}=\frac{1}{\left|J_{c}\right|}\sum_{I\in J_{c}}E_{v}(I)$$

where $T_{c}$ denotes the set of textual descriptions corresponding to the c-th defect category, and $J_{c}$ denotes the set of image samples corresponding to the c-th defect category. The textual prototype is used to represent the semantic center of this defect category, while the visual prototype is used to represent the typical appearance distribution of this defect category in the image space. Together, they constitute category-level cross-modal reference information.

For the candidate region fused representation $z_{i}^{f}$, its similarity to the prototype of the c-th defect category is defined as

(22) $$s_{i,c}^{proto}=\rho \cos(z_{i}^{f},p_{c})+(1-\rho )\cos(z_{i}^{f},q_{c})$$

where ρ denotes the fusion weight of the textual prototype and visual prototype, and cos(⋅) denotes cosine similarity. This similarity is used to measure the consistency between a candidate region and different defect category centers.

To enhance the compactness between the candidate region representation and the prototype of its true category, this paper adopts the prototype constraint loss:

(23) $$L_{proto}=-\frac{1}{N_{r}}\sum_{i=1}^{N_{r}}\log\frac{\exp(s_{i,y_{i}}^{proto}/\tau_{p})}{\sum_{c=1}^{C}\exp(s_{i,c}^{proto}/\tau_{p})}$$

where $y_{i}$ denotes the ground-truth category corresponding to the candidate region, C denotes the number of defect categories, and $\tau_{p}$ denotes the prototype temperature coefficient. This loss encourages the candidate region representation to approach the prototype of its true category while moving away from similar but mismatched category prototypes.

Combined with the detection head output, the model completes defect category prediction and bounding box regression. The detection-stage loss is defined as

(24) $$L_{det}=L_{cls}+\lambda_{reg}L_{reg}+\lambda_{proto}L_{proto}$$

where $L_{cls}$ denotes the classification loss, $L_{reg}$ denotes the bounding box regression loss, $L_{proto}$ denotes the prototype constraint loss, and $\lambda_{reg}$ and $\lambda_{proto}$ are weight coefficients.

The prototype constraint does not replace the detection head but provides additional category-center constraints for detection representations. By combining textual and visual prototypes, the model obtains more stable category boundaries in the cross-modal space, improving discrimination of similar categories, few-shot defects, and semantics-dependent defects.

### 3.5. Knowledge Rule Verification and Posterior Re-Scoring

Substation equipment has clear spatial layouts and topological relationships. Some defects depend not only on local appearance but also on equipment area, spatial location, and operation–maintenance rules. For example, bird nests usually appear on structures, crossbeams, or high supporting areas, while similar targets in unreasonable locations are more likely to be background interference. Therefore, this paper introduces a knowledge rule verification module for posterior consistency judgment of candidate results.

For candidate defect target *i*, the neural perception score is defined as

(25) $$s_{i}^{neu}=\omega_{1}p_{i}+\omega_{2}s_{i,c}^{proto}+\omega_{3}s_{i}^{align}$$

where $p_{i}$ denotes the confidence score of the detection head, $s_{i,c}^{proto}$ denotes the similarity between the candidate region and the category prototype, $s_{i}^{align}$ denotes the local image–text alignment score, and $\omega_{1}$, $\omega_{2}$, and $\omega_{3}$ are weight coefficients.

The rule verification branch constructs rule features $h_{i}^{rule}$ according to the candidate target category, spatial location, associated equipment area, and topological relationship, and its rule score is expressed as

(26) $$s_{i}^{rule}=\sigma (W_{r}h_{i}^{rule}+b_{r})$$

where $W_{r}$ and $b_{r}$ are the parameters of the rule evaluation function. Subsequently, a soft re-scoring strategy is adopted to fuse the neural perception score and the rule verification score:

(27) s^i=γisineu+(1−γi)sirule

where $\gamma_{i}$ denotes the dynamic fusion weight, which is defined as

(28) $$\gamma_{i}=\sigma \left(W_{\gamma }\left[s_{i}^{neu},s_{i}^{rule},u_{i}\right]+b_{\gamma }\right)$$

where $u_{i}$ denotes the comprehensive uncertainty estimate of the candidate region. This mechanism can retain candidate results when the visual evidence is sufficient and consistent with rules, while reducing their confidence when the visual appearance is similar but the spatial logic is unreasonable.

The knowledge rule verification module mainly reduces false positives caused by logical conflicts and improves result reliability and interpretability, rather than simply increasing average detection accuracy. Soft re-scoring, instead of hard filtering, also reduces the adverse impact of incomplete or noisy rules on recall.

### 3.6. Model Training and Inference Procedure

This paper adopts staged training with joint fine-tuning. Vertical-domain image–text pretraining first obtains visual and text encoders for substation defect scenarios. Then, bidirectional cross-modal mapping is trained for region–text alignment, followed by joint training of the detection head and prototype constraint module for localization and category discrimination. Finally, knowledge rule verification is introduced for posterior re-scoring and consistency constraints.

The overall training objective of the model is defined as

(29) $$L_{total}=\eta_{1}L_{pre}+\eta_{2}L_{map}+\eta_{3}L_{det}+\eta_{4}L_{rule}$$

where $L_{pre}$, $L_{map}$, $L_{det}$, and $L_{rule}$ denote the vertical-domain pretraining loss, cross-modal mapping loss, detection discrimination loss, and rule consistency loss, respectively, and $\eta_{1}$ to $\eta_{4}$ are weight coefficients.

During inference, the system extracts visual features, generates candidate regions, and performs cross-modal recognition using textual features and category prototypes. Equipment topology and operation–maintenance rules are then used for posterior re-scoring, producing bounding boxes, defect categories, confidence scores, associated equipment, rule verification results, and interpretation information.

Since image–text pretraining is completed offline, textual prototypes, category semantic vectors, and the rule base can be pre-encoded and cached. Thus, online inference mainly involves visual feature extraction, candidate generation, cross-modal matching, and lightweight rule re-scoring, reducing computational overhead.

## 4. Experiment and Results Analysis

### 4.1. Experimental Platform and Parameter Settings

The experiments were conducted on Ubuntu 20.04 (Canonical Ltd., London, UK) using PyTorch 2.1 (Linux Foundation, https://pytorch.org) and CUDA 11.8 (NVIDIA Corporation, Santa Clara, CA, USA; https://developer.nvidia.com/cuda-toolkit (accessed on 8 June 2026)), with an Intel Xeon Silver 4314 CPU (Intel Corporation, Santa Clara, CA, USA), 128 GB of memory, and two NVIDIA RTX 3090 GPUs (NVIDIA Corporation, Santa Clara, CA, USA). The model was trained with AdamW optimizer (implemented in PyTorch 2.1), using an initial learning rate of $1\times 10^{-4}$, weight decay of $1\times 10^{-2}$, $\beta_{1}=0.9$, $\beta_{2}=0.999$, a batch size of 16, and 120 epochs. The learning rate was decayed by 0.1 at the 80th and 100th epochs. All images were resized to 640×640 pixels, with random flipping, brightness perturbation, contrast adjustment, slight blurring, and occlusion simulation applied for data augmentation.

For model configuration, the visual encoder used a CLIP Vision Transformer (OpenAI, San Francisco, CA, USA) backbone with a multi-scale feature pyramid, and the text encoder used a Transformer branch with a maximum text length of 64. The image–text and knowledge embedding dimensions were 512 and 256, respectively. The loss weights for image–text contrastive learning, attribute supervision, environmental consistency, prototype constraint, local alignment, and rule consistency were 1.0, 0.5, 0.3, 0.2, 0.8, and 0.4. The uncertainty-aware gating module used a two-layer perceptron with a hidden dimension of 256, and the logical re-scoring threshold was 0.57.

Each experiment was repeated three times and averaged. The evaluation metrics included Precision, Recall, F1-score, mAP@0.5, mAP@0.5:0.95, false-positive rate, and logical-conflict false positives. For fair comparison, all baselines used the same dataset split, input resolution, and metrics, while You Only Look Once version 9 (YOLOv9), You Only Look Once version 10 (YOLOv10), Real-Time Detection Transformer (RT-DETR), and Collaborative Hybrid Assignments Training Detection Transformer (Co-DETR) followed their official implementations and default training strategies.

The model inference efficiency was evaluated on the same hardware platform. With an input size of 640×640 and a batch size of 1, the proposed model had 52.6 M parameters and a size of 201.8 MB. After 200 warm-up iterations and 1000 repeated runs, the average inference latency was 37.5 ms per image, corresponding to 26.7 frames per second, with a peak GPU memory usage of 4.8 GB. Since image–text pre-training was completed offline, online inference mainly involved visual feature extraction, cross-modal matching, and lightweight rule re-scoring.

### 4.2. Dataset Setup and Task Description

The experimental data were collected from camera-based inspection and monitoring in real substations, considering only visible-light images. After quality screening, classification, and expert review, the images were annotated with defect categories and bounding boxes. The data included inspection images, multi-granularity defect text descriptions, and equipment topology rules for visual detection, cross-modal semantic alignment, and posterior logical verification.

The dataset contained 11,842 images, including 2916 normal samples and 8926 defect samples. Six defect categories were considered: bird nests, suspended foreign objects, insulator damage, metal fitting corrosion, abnormal switch status, and equipment surface abnormalities. The data covered sunny, cloudy, strong illumination, fog, occlusion, small-target, and complex-background scenarios. The dataset was split into training, validation, and test sets at 70%, 10%, and 20% using stratified splitting, as shown in Table 1.

The textual semantic data were generated through a template-driven semi-automatic process. Domain experts defined templates based on defect categories, equipment types, defect attributes, spatial locations, and equipment status, which were then expanded using image labels and inspection scenarios and reviewed by experts. Each defect sample was associated with 2–4 text descriptions covering category, attribute, spatial relationship, and status information, yielding 36,714 descriptions. Equipment topology rules were constructed from spatial relationships, typical defect locations, and operation and maintenance experience for rule verification.

**Figure 3:**
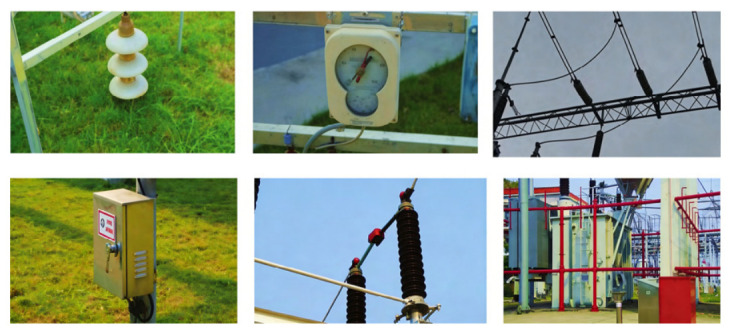
Substation Normal State Dataset.

**Figure 4:**
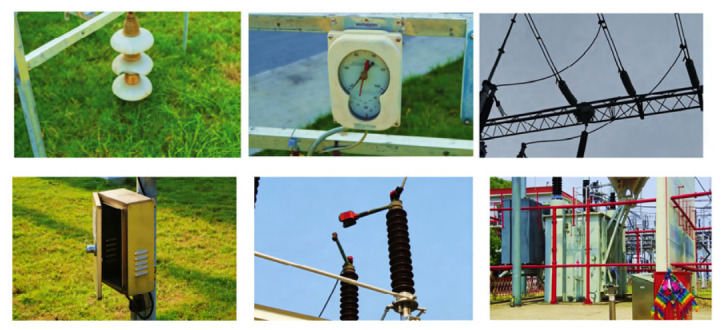
Substation Defect Status Dataset.

Figure 3 and Figure 4 show representative normal-state and defect-state samples, respectively. Based on these data, the experiments focus on overall detection performance, key module contribution, and robustness and interpretability in complex scenarios.

### 4.3. Comparison with Mainstream and Recent Detection Models

To verify overall detection performance, the proposed method is compared with Faster Region-based Convolutional Neural Network (Faster R-CNN), You Only Look Once version 8 (YOLOv8), YOLOv9, YOLOv10, Deformable Detection Transformer (Deformable DETR), RT-DETR, Co-DETR, CLIP + YOLOv8, and Text-Fusion Detector. All models use the same dataset split, input resolution, and evaluation metrics. The results are shown in Table 2.

As shown in Table 2, recent detectors such as YOLOv9, YOLOv10, RT-DETR, and Co-DETR outperform traditional baselines, showing that stronger visual structures improve substation defect identification. Co-DETR performs best among visual detectors, with mAP@0.5 and mAP@0.5:0.95 of 89.1% and 66.0%.

**Figure 5:**
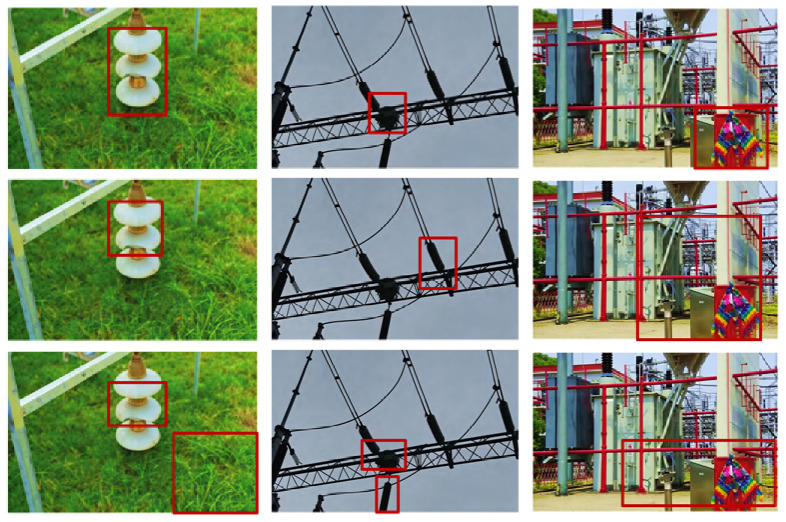
Effects of different defect detection methods.

The proposed method achieves state-of-the-art performance, reaching 90.8% mAP@0.5 and 68.7% mAP@0.5:0.95—improving by 1.7%/2.7% over Co-DETR and 2.1%/3.4% over RT-DETR, respectively. These results confirm that vertical-domain image–text pre-training, region-level cross-modal mapping, prototype constraints, and knowledge rule verification enhance semantic modeling and suppress unreasonable false detections. Figure 5 provides visual evidence: the proposed method better localizes real defects and reduces redundant predictions in complex scenarios, with red squares highlighting baseline false positives and missed defects that the proposed method addresses.

### 4.4. Performance Analysis of Detection for Each Defect Category

To further analyze category adaptability, Table 3 presents the AP@0.5 results of YOLOv8, CLIP + YOLOv8, and the proposed method on six defect categories, while Figure 5 shows the corresponding visual comparisons.

**Figure 6:**
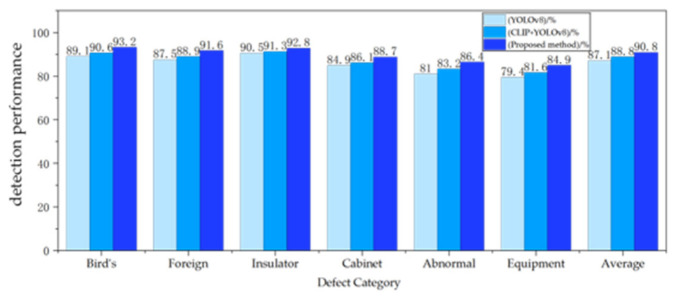
Bar chart comparing the performance of various detection methods under different defect categories.

As shown in Table 3 and Figure 6, the proposed method achieves the best AP@0.5 across all six defect categories. Compared with YOLOv8, it shows larger gains for bird nests, suspended foreign objects, abnormal switch status, and equipment surface abnormalities, with improvements of 5.4% and 5.5% for the latter two categories. This indicates that cross-modal semantic supplementation and rule constraints are especially effective for defects relying on spatial location, equipment context, and semantic information.

### 4.5. Analysis of Ablation Test Results

To verify the contribution of each component module to the overall performance, this paper designs a step-by-step ablation experiment. The experimental results are shown in Table 4, where “√” indicates that the module is used and “×” indicates that it is excluded.

As shown in Table 4, the vertical-domain pre-training module improves mAP by 1.1%, indicating that image–text joint pre-training for power scenarios enhances the quality of basic representations. After introducing bidirectional cross-modal mapping, mAP further increases by 1.4%, demonstrating the key role of fine-grained image–text interaction in complex defect semantic recognition. Adding prototype constraints brings another 0.5% improvement, showing that category prototypes enhance discrimination between semantically similar categories.

The neuro-symbolic reasoning module provides a certain improvement in mAP@0.5 and F1-score, but its effect on average detection accuracy is relatively limited. Its main role is to verify the logical consistency of candidate results and suppress false positives that appear visually reasonable but conflict with equipment topology or spatial constraints. Overall, all four modules contribute positively and produce cumulative gains, indicating that the proposed framework forms effective synergy in feature modeling, semantic alignment, and logical reasoning rather than simply stacking individual modules.

### 4.6. Ablation and Sensitivity Analysis

To verify the contribution of each component module to the overall performance, this paper designed a step-by-step ablation experiment, and the results are shown in Table 5, where “√” means the loss term is applied and “×” means it is removed.

As shown in Table 5, removing the local alignment loss causes the most significant performance degradation, indicating that region–text semantic alignment is critical for complex defect recognition. Removing attribute supervision, prototype constraints, and rule consistency loss also reduces performance to varying degrees, showing their roles in fine-grained attribute representation, similar-category discrimination, and logical consistency enhancement, respectively. When environmental consistency loss is removed, mAP@0.5 decreases from 90.8% to 90.0%, suggesting that this regularization improves feature stability under different visual conditions. Its environmental adaptability is further verified in Section 4.7.

Considering the use of multi-granularity defect text descriptions, this paper further evaluates the model’s dependence on text resources. Five settings are tested: full text, 50% retained text, 25% retained text, category-only text, and no text input. Full text includes category, attribute, location, and status descriptions; category-only text retains only defect categories; and no text removes the cross-modal semantic branch, degrading the model into a vision-dominant detector. This experiment simulates practical deployment with coarse-grained or incomplete text resources, and the results are shown in Table 6.

As shown in Table 6, as text resources decrease, the model performance shows a gradual downward trend, indicating that fine-grained semantic descriptions help improve the stability of defect recognition. It is worth noting that when only category-level text is retained, the model still achieves an mAP@0.5 of 88.9%, which is higher than the 87.6% obtained under the no-text setting. This indicates that coarse-grained semantic information can still provide effective category guidance. Overall, the proposed framework can benefit from fine-grained text resources, but it does not completely rely on densely manually constructed text descriptions and still maintains a certain degree of applicability under limited-text conditions.

### 4.7. Robustness Analysis of Complex Scenarios

**Figure 7:**
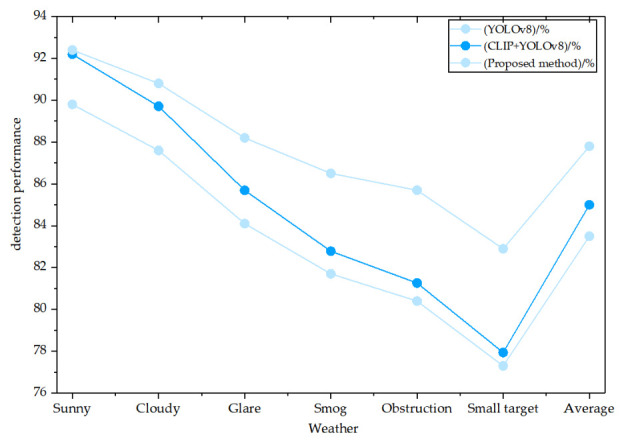
Performance comparison line chart of various detection methods in different complex scenarios.

Considering that substation inspection scenarios are usually accompanied by problems such as illumination changes, weather interference, occlusion, and small targets, this paper further tests the model on different complex-environment subsets. Table 7 and Figure 7 present the mAP@0.5 results of YOLOv8, CLIP + YOLOv8, and the proposed method under different scenarios.

The experimental results show that the proposed method achieves the highest mAP@0.5 on all complex-scenario subsets, with more obvious advantages under strong illumination, fog, occlusion, and small-target conditions. Compared with YOLOv8, it improves performance by 4.8%, 5.3%, and 5.6% in fog, occlusion, and small-target scenarios, respectively. This indicates that cross-modal semantic supplementation and knowledge rule verification provide additional criteria when image quality decreases or target representation is insufficient, thereby improving robustness in complex environments.

It should be noted that this experiment evaluates environmental adaptability under the visual conditions covered by the current measured dataset. Although the results indicate improved robustness in representative complex scenarios, further validation with longer-period, multi-site, and continuously collected field data will be conducted in future work to evaluate the performance of the proposed framework in online inspection applications.

### 4.8. Analysis of False-Positive Suppression and Inference Effects

To further evaluate the reliability of defect prediction results under sample imbalance and complex visual conditions, this paper counts the changes in false positives before and after introducing the neuro-symbolic reasoning module. The results are shown in Table 8.

As shown in Table 8, although the neuro-symbolic reasoning module brings limited improvement in average detection metrics, it significantly reduces false positives. After introducing this module, total false positives decrease from 198 to 146, logical-conflict false positives decrease from 79 to 31, and the overall false-positive rate decreases from 8.7% to 6.1%. The 60.8% reduction in logical-conflict false positives indicates that soft logical re-scoring can suppress predictions inconsistent with equipment topology, spatial locations, or operation and maintenance rules while retaining valid candidates, thereby improving engineering reliability and interpretability. Its stability under incomplete and noisy rules will be further analyzed in Table 9.

### 4.9. Cross-Modal Interpretability Analysis

**Figure 8:**
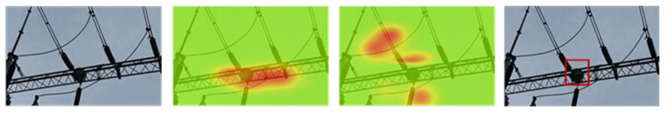
Visualization of Attention Response under Cross-Modal Text Guidance.

In addition to overall performance and false-positive control, this paper analyzes the role of the cross-modal feature mapping module in defect localization. Figure 8 shows the model’s attention response distributions under different defect text inputs. In the figure, the red square marks the detected defect location, and the color maps (from green to red) represent attention intensity, with red indicating the highest response. When descriptions such as a bird nest located near the framework beam or a suspended foreign object hanging near the conductor are input, the high-response regions accurately focus on the corresponding defects, while attention to irrelevant backgrounds is reduced. This indicates that the proposed bidirectional dynamic cross-modal mapping mechanism effectively establishes fine-grained correspondences between local image regions and textual semantics, thereby improving discrimination in complex defect scenarios.

Combined with the detection results in Figure 3, cross-modal semantics not only provide additional information for classification but also guide the visual encoder to focus on more discriminative regional features. This visually verifies the effectiveness of the proposed cross-modal semantic enhancement strategy.

## 5. Conclusions

This paper proposes a multimodal defect perception method based on cross-modal feature mapping for complex substation inspection scenarios. By integrating equipment images, defect text descriptions, and topology knowledge, a unified vertical-domain pre-training–cross-modal mapping–hierarchical reasoning framework is constructed to improve defect monitoring accuracy, robustness, and interpretability. Specifically, vertical-domain adaptive pre-training enhances representations of equipment categories, defect attributes, and scene semantics. Image–text bidirectional cross-modal mapping improves semantic alignment and complex defect discrimination, while neuro-symbolic reasoning integrates spatial relationships and topology knowledge for logical consistency verification and false-positive suppression. Experimental results show that the proposed method outperforms typical single-modal detection and simple multimodal fusion methods in overall performance, complex-scenario robustness, and false-positive control. Ablation and visualization analyses verify the effectiveness of key modules and demonstrate the rationality of cross-modal semantic guidance and knowledge constraints, indicating their potential for intelligent substation equipment monitoring.

## Figures and Tables

**Table 1:** Distribution of normal and defect samples in the dataset.

Subset	Normal Samples	Defect Samples	Total Samples	Normal Ratio	Defect Ratio
Training set	2041	6248	8289	24.62%	75.38%
Validation set	292	893	1185	24.64%	75.36%
Test set	583	1785	2368	24.62%	75.38%
Total	2916	8926	11,842	24.62%	75.38%

**Table 2:** Comparison of overall detection performance of different methods.

Method	Precision/%	Recall/%	F1/%	mAP@0.5/%	mAP@0.5:0.95/%
Faster R-CNN	84.6	80.1	82.3	83.7	59.2
YOLOv8	87.9	83.8	85.8	87.1	63.4
YOLOv9	88.4	84.6	86.5	88.0	64.5
YOLOv10	88.8	85.0	86.9	88.5	65.1
Deformable DETR	87.2	84.5	85.8	87.6	64.1
RT-DETR	88.6	85.3	86.9	88.7	65.3
Co-DETR	89.0	85.8	87.4	89.1	66.0
CLIP + YOLOv8	88.9	85.2	87.0	88.8	65.5
Text-Fusion Detector	89.3	85.7	87.5	89.2	66.1
Proposed method	91.1	87.8	89.4	90.8	68.7

**Table 3:** Comparison of detection performance for each defect category.

Defect Category	YOLOv8/%	CLIP + YOLOv8/%	Proposed Method/%
Bird’s Nest	89.1	90.6	93.2
Foreign object suspension	87.5	88.9	91.6
Insulator damage	90.5	91.3	92.8
Cabinet door opening and closing abnormally	84.9	86.1	88.7
Abnormal opening and closing of disconnecting switch	81.0	83.2	86.4
Equipment surface abnormality	79.4	81.6	84.9
Average	87.1	88.8	90.8

**Table 4:** Module ablation experimental results.

Model Number	Vertical Pre-Training	Bidirectional Cross-Modal Mapping	Prototype Constraints	Neurosymbolic Reasoning	mAP@0.5/%	mAP@0.5:0.95/%	F1/%
A	×	×	×	×	87.1	63.4	85.8
B	√	×	×	×	88.2	64.8	86.7
C	√	√	×	×	89.6	66.5	88.0
D	√	√	√	×	90.1	67.2	88.6
E	√	√	√	√	90.8	68.7	89.4

**Table 5:** Loss term ablation experiment results.

Model Settings	$L_{attr}$	$L_{inv}$	$L_{proto}$	$L_{loc}$	$L_{rule}$	mAP@0.5/%	F1/%
Complete model	√	√	√	√	√	90.8	89.4
Remove attribute supervision	×	√	√	√	√	90.1	88.8
Remove environmental consistency	√	×	√	√	√	90.0	88.7
Remove prototype constraints	√	√	×	√	√	90.2	88.9
Remove local alignment	√	√	√	×	√	89.5	88.0
Remove rule consistency	√	√	√	√	×	90.1	88.9

**Table 6:** Performance comparison under different text resource settings.

Text Setting	Description	mAP@0.5/%	mAP@0.5:0.95/%	F1/%
Full text	Category + attribute + location + status	90.8	68.7	89.4
50% text	50% retained descriptions	90.2	67.9	88.8
25% text	25% retained descriptions	89.5	66.8	88.1
Category-only text	Defect-category descriptions only	88.9	65.9	87.5
No text	Visual-dominant detector	87.6	64.2	86.3

**Table 7:** Robustness evaluation on field inspection subsets with different visual conditions.

Method	Sunny	Cloudy	Glare	Smog	Obstruction	Small Target	Average
YOLOv8/%	89.8	87.6	84.1	81.7	80.4	77.3	83.5
CLIP + YOLOv8/%	90.7	88.9	86.0	83.9	82.8	80.4	85.5
Proposed method/%	92.4	90.8	88.2	86.5	85.7	82.9	87.8

**Table 8:** False-positive suppression analysis under neuro-symbolic reasoning.

Index	No Reasoning Used	Using Reasoning	Range of Change
Total number of false-positive samples	198	146	−26.3%
False Detection Number of Logical Conflicts	79	31	−60.8%
False positives for bird nests	46	29	−37.0%
False detections of foreign object suspension	58	40	−31.0%
False-positive rate (FPR)/%	8.7	6.1	−2.6 pct

**Table 9:** Robustness analysis under incomplete and noisy rule settings.

Rule Setting	mAP@0.5/%	F1/%	Total FP	Logical-Conflict FP
Full rules	90.8	89.4	146	31
50% rules retained	90.4	88.9	163	47
25% rules retained	90.2	88.7	177	61
20% noisy rules	90.3	88.8	166	49
No rules	90.1	88.6	198	79

## Data Availability

No new data were created or analyzed in this study.
